# One‐Step 3D Printing of Heart Patches with Built‐In Electronics for Performance Regulation

**DOI:** 10.1002/advs.202004205

**Published:** 2021-03-02

**Authors:** Masha Asulin, Idan Michael, Assaf Shapira, Tal Dvir

**Affiliations:** ^1^ The Shmunis School of Biomedicine and Cancer Research Faculty of Life Sciences Tel Aviv University Tel Aviv 6997801 Israel; ^2^ Department of Materials Science and Engineering Faculty of Engineering Tel Aviv University Tel Aviv 6997801 Israel; ^3^ The Center for Nanoscience and Nanotechnology Tel Aviv University Tel Aviv 6997801 Israel; ^4^ Sagol Center for Regenerative Biotechnology Tel Aviv University Tel Aviv 6997801 Israel

**Keywords:** 3D printing, bioinks, cardiac tissue engineering, ECM hydrogels, electronics

## Abstract

Three dimensional (3D) printing of heart patches usually provides the ability to precisely control cell location in 3D space. Here, one‐step 3D printing of cardiac patches with built‐in soft and stretchable electronics is reported. The tissue is simultaneously printed using three distinct bioinks for the cells, for the conducting parts of the electronics and for the dielectric components. It is shown that the hybrid system can withstand continuous physical deformations as those taking place in the contracting myocardium. The electronic patch is flexible, stretchable, and soft, and the electrodes within the printed patch are able to monitor the function of the engineered tissue by providing extracellular potentials. Furthermore, the system allowed controlling tissue function by providing electrical stimulation for pacing. It is envisioned that such transplantable patches may regain heart contractility and allow the physician to monitor the implant function as well as to efficiently intervene from afar when needed.

## Graphical abstract

The one‐step 3D printing of cardiac patches with built‐in soft and stretchable electronics is reported. The tissue is printed using three distinct bioinks for the cells and the conducting and dielectric parts of the electronics. The electrodes within the printed patch can monitor the function of the engineered tissue, and control tissue function by providing electrical stimulation for pacing.

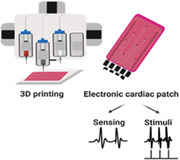

## 1 Introduction

Cardiac tissue engineering has emerged as a promising therapy to regenerate the diseased heart. In this approach, rather than simply introducing cells into the diseased area to repopulate the injured heart and restore function, the cells are seeded in or onto 3D biomaterials prior to transplantation. These materials mimic the extracellular matrix (ECM) and serve as temporary scaffolds supporting the cells and promoting their reorganization into a functional cardiac patch.^[^ ^1^, ^2^, ^3^, ^4^, ^5^, ^6^, ^7^, ^8^ ^]^

Recently, the field of 3D printing has evolved to allow precise placement of cells in the desired location within the scaffold and even for the engineering of volumetric structures such as whole organs.^[^ ^9^, ^10^, ^11^, ^12^ ^]^ In recent studies, different approaches were developed to control blood vessel orientation within the patches.^[^ ^13^, ^14^, ^15^, ^16^ ^]^ Specifically, we have developed an approach where patient‐specific cells, including induced pluripotent stem cell (iPSC)‐derived cardiomyocytes, endothelial cells, and pericytes, were 3D printed together with a patient‐specific omentum ECM hydrogel to create a personalized vascularized heart patch.^[^ ^17^ ^]^ The printed patch was functional as demonstrated by its ability to spontaneously propagate the electrical signal throughout the tissue. However, once the patches are engineered, in vitro assessment of their quality in terms of electrical activity, without affecting their performance, is limited.^[^ ^18^ ^]^ This may lead to the implantation of heart patches with limited or no potential to regenerate the diseased heart. The cells may be electrophysiologically dysfunctional, jeopardizing the efficacy of the treatment. More importantly, the ability to monitor and control the performance of these patches following implantation is completely lost.

Recently, our group has suggested a new concept in tissue engineering, where a planar electronic system is fabricated by lithography and in a second step integrated within the 3D scaffolds.^[^ ^19^, ^20^ ^]^ In a third step, cardiac cells are seeded on the electronics‐integrated scaffold to create the electronic patch. The electronic components are allowed to record tissue function, activate the cells by providing electrical stimulation, and release different types of drugs within the cellular microenvironment. However, in this case, the lithography‐made electronic system was significantly stiffer than living tissue. Such a mismatch in the mechanical properties may induce an immune response, which in turn may lead to rejection of the patch.^[^ ^21^ ^]^ Additionally, the electronic system was two dimensional (2D) and in order for it to provide data from a thick cardiac patch, the entire hybrid construct was folded after cell seeding.

**Figure 1:**
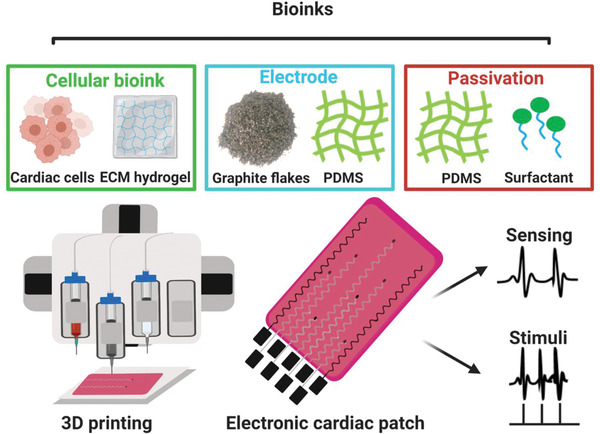
Schematics of the concept. Three distinct bioinks were produced: A bioink composed of cardiac cell‐containing ECM hydrogel to form the tissue, a bioink composed of the conducting material of the electronics, and a bioink containing the dielectric material which passivates the electrodes, leaving open conducting pads for point‐sensing and stimulation. The three bioinks were 3D printed together to engineer the electronic cardiac patch. The electronics within the patch can provide sensing of extracellular signals and stimulation for pacing.

Here, we report the one‐step 3D printing of heart patches with built‐in soft electronics. To this end, three distinct bioinks were used (**Figure** **1**). The cellular bioink formed the engineered tissue and contained an ECM‐based hydrogel mixed with neonatal rat ventricular cardiac cells. We have previously shown the potential of omentum ECM hydrogel to support cell growth and tissue formation.^[^ ^22^ ^]^ Moreover, we have demonstrated the advantages of omentum‐based hydrogel for 3D printing of vascularized cardiac tissues and whole hearts.^[^ ^9^, ^17^ ^]^ The ECM hydrogel is a weak hydrogel at room temperature and becomes stiffer at 37 °C. A second bioink contained the conductive material of the electronic system and was composed of graphite flakes suspended in liquid polydimethylsiloxane (PDMS) at a high concentration. This component formed the electrodes and allowed recording and stimulation of the engineered tissue. Finally, the third bioink contained the dielectric material covering the electrodes. This component contained liquid PDMS and was used to passivate the entire electrode, leaving exposed conducting pads at the end of each electrode. This setup allowed to control the location of the sensing/stimulation area, reducing noises that may have been collected along the electrodes. Additionally, to better interface between the hydrophilic part of the printed patch (i.e., the cell‐containing hydrogel) and the hydrophobic parts (i.e., electronic components), a non‐ionic surfactant, span 80, was added to the PDMS. The three bioinks were loaded into three extrusion cartridges within the 3D printer, allowing simultaneous printing of the electronic cardiac patch for future application of drug testing or for treating the infarcted heart (Figure 1).

## 2 Results and Discussion

**Figure 2:**
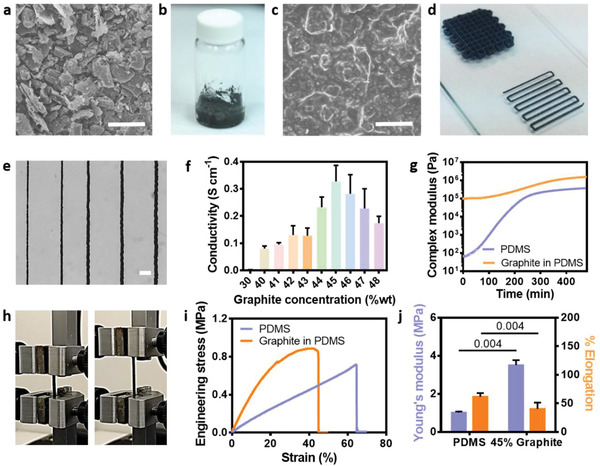
Electrode formulation and characterization. a) Scanning electron microscopy (SEM) image of graphite flakes, which compose the conducting part of the electrodes. Scale bar = 50 µm. b) Macroscopic view of the bioink, consisting of the graphite flakes in PDMS. c) SEM image of the conducting bioink. Scale bar = 50 µm. d,e) The bioink can be easily printed to form fine structures (d) with various widths (e). Scale bar = 1 mm. f) Electrical conductivity of the printed electrodes (n = 3). g) The complex viscosity of PDMS or 45% graphite flakes in PDMS (n = 3) tested using a rheometer. h) Stretching the graphite dog‐bone‐shaped samples by a mechanical tester. Left: before stretching; Right: after stretching. i) Engineering stress versus strain graph of PDMS or graphite flakes in PDMS. j) Young's modulus and elongation of the PDMS or graphite flakes in PDMS (n = 4).

To fabricate the conductive part of the electronics, we decided to use graphite flakes with an average diameter of 14.5±9.5 µm and circularity of 0.7±0.1 µm (**Figure** **2**a and Figure S1a,b, Supporting Information). The dried particles were mixed with PDMS (Figure 2b) at different concentrations ranging between 30–48% (wt) to create a viscous printable paste (Figure 2c,d), allowing to control the width of the extruded material, reaching ≈140 µm wide electrodes (Figure 2e and Figure S1c, Supporting Information). The conductivity of the different compositions after curing at 37 °C was measured using a high‐resolution multimeter, revealing the highest conductivity for the 45% blend (Figure 2f). This may be attributed to the high viscosity of the paste above 45%, leading to discontinuous printing. As shown, the incorporation of the graphite within the PDMS increased the viscosity of the hybrid material compared to pure PDMS (Figure 2g).

We next sought to evaluate the mechanical properties of the graphite mixture, which was designed to serve as an electrode, using a mechanical tester. As shown, the two ends of the dog‐bone shaped conductive material were held and stretched by the mechanical tester (Figure 2h). Figure 2i shows a representative stress‐strain behavior of the material, demonstrating its robustness up to 50% and elasticity below 20% strain. Further analysis of the mechanical properties revealed that although integration of the graphite within the PDMS increased the Young's modulus and reduced elongation (Figure 2j), the electrodes still maintained suitable properties for serving as components in cardiac patches, as the heart expands approximately 20% during normal function.^[^ ^23^, ^24^ ^]^

**Figure 3:**
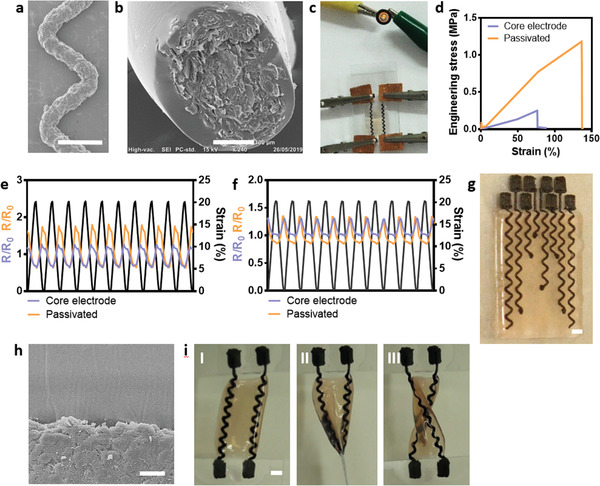
Electrode passivation, function, and integration. a) SEM image of printed pristine serpentine‐shape electrode. Scale bar = 1 mm. b) Cross‐section of the passivated electrode. Scale bar = 100 µm. c) Using the electrodes for activation of a LED. d) Engineering stress versus strain of the pristine and passivated serpentine electrodes. e) The resistance and strain during the last 10 cycles of 1000 stretching cycles. f) The resistance and strain during the last 10 cycles of 1000 bending cycles. g) The printed cardiac patch containing 8 electrodes. Scale bar = 2 mm. h) Cross‐section of the patch reveals the interface of the passivation layer (upper part) and the hydrogel with cells (lower part). Scale bar = 20 µm. i) Photographs of the electrodes I) before twisting, II) during twisting of 90°, and III) after twisting at 180°. Scale bar = 2 mm.

The serpentine structure of lithography‐fabricated electrodes was previously shown to enable stretching without compromising its function.^[^ ^20^, ^25^ ^]^ We therefore printed electrodes with such a structure (**Figure** **3**a). Passivation of the electrodes is essential for accurate spatial sensing and stimulation and for reducing noise. Therefore, PDMS layers were printed around the graphite paste, resulting in complete coverage (Figure 3b and Figure S2a–d, Supporting Information). Application of voltage through the electrodes enabled activation of a light‐emitting diode (LED) (Figure 3c), with minimal voltage loss (Figure S2e, Supporting Information). Furthermore, long‐term repetitive electrical stimulation through the electrodes (3V at 1Hz for 6h) did not affect their resistance (Figure S2f, Supporting Information). To assess the degradation of the electrodes and its effect on their performance, we incubated the electrodes for two months in a culture medium and for 7 days at 37 °C in a medium supplemented with collagenase. In both tests, the performance of the electrodes with or without passivation was not affected (Figure S3a,b, Supporting Information). As PDMS may absorb molecules to its surface, a slight accumulation of molecules from the culture medium could be seen on the surface of the electrodes. (Figure S3c–f, Supporting Information). We next sought to evaluate the mechanical properties of the passivated serpentine electrode. Interestingly, the passivated electrode could stretch to 135%, significantly longer than the pristine electrode and with a Young's modulus of 0.95±0.2 MPa (Figure 3d, Figure S3g, and Movie S1, Supporting Information). This may be attributed to the tight interaction between the PDMS in the electrode and the PDMS of the passivation layer, which strengthened the entire structure. We next investigated whether changes in resistivity occur during 1000 cycles of mechanical deformations. The electrodes function was steady throughout the test with a slight increase at the first cycles (Figure S4a,b, Supporting Information). Such an increase is typical to flakes‐consisting hybrid materials.^[^ ^26^, ^27^, ^28^ ^]^ As shown, in the last 10 cycles of the cyclic stretching (Figure 3e, and Movie S2, Supporting Information) and cyclic bending (Figure 3f and Movie S3, Supporting Information), the resistance of the electrodes fluctuated around the initial value, indicating that their overall function was not impaired. SEM images validated electrode integrity following multiple stretches (Figure S4c–f, Supporting Information). Moreover, the hybrid printed material containing both the electronics and the ECM hydrogel was exposed to cyclic stretching. As shown, the performance of the electrodes and their integrity were not affected (Figure S5, Supporting Information). Overall, these results imply that the printed electronic system can withstand the expansion of the heart and can operate properly.

The ability to print the passivated electrodes within an ECM‐based hydrogel was next tested. This required using three printheads in parallel. A layer of cardiac cell‐containing hydrogel was printed with serpentine grooves for the printed electronics. Next, the electronics were printed, including the bottom passivation layer, the conducting material of the electrode, and the upper passivation layer (Figure 3g). The latter was designed to leave open pads at the end of the conducting material, which serve as the electrode‐cell interface. Cell‐containing ECM‐based hydrogel layer was then printed on top of this construct, creating a close interaction between the electronics and hydrogel (Figure 3h and Figure S6a,b, Supporting Information). As shown, the printed electronic tissue was soft and robust and could withstand twisting and stretching (Figure 3i and Movie S4, Supporting Information).

**Figure 4:**
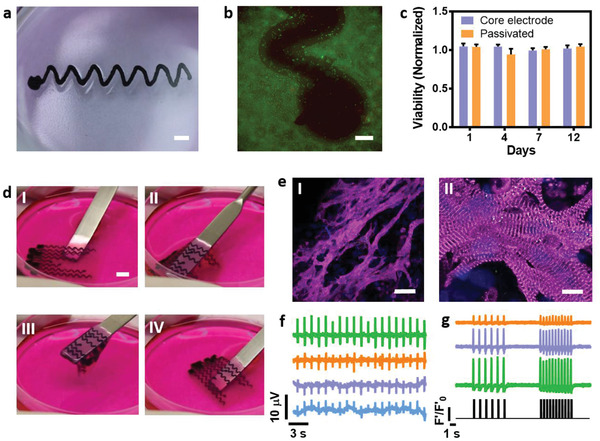
Viability, morphology, and patch function. a) Passivated electrode printed in a tissue culture plate. Cardiac cells were then seeded in the plate. Scale bar = 2 mm. b) Live/dead assay of cardiac cells on and near the electrode on day 12. Scale bar = 100 µm. c) Cardiac cell viability as assessed by Presto blue assay after culture with pristine or passivated electrodes. Cell viability is normalized to cardiac cell culture without electrodes (n = 3). d) The 3D printed patch is flexible and soft. I–IV are pictures of the patch lifted up and returned to the culture medium. Scale bar = 6 mm. e) Immunostaining for sarcomeric actinin (pink) and nuclei (blue) of a 12‐day cardiac patch. Scale bar: (I) = 50 µm and (II) = 10 µm. f) Simultaneous recording of extracellular potentials of cardiac cells from 4 distinct locations. g) Calcium transients from 3 distinct areas within the patch after pacing (7 V at 1 and 2 Hz) using the printed electrodes. The pacing pattern is in the lower part of the figure.

To evaluate the toxicity of the printing process and electrodes, electrodes were printed into a cell culture plate. Subsequently, cardiac cells, isolated from the ventricles of neonatal rats, were seeded on the electrode (both on the dielectric material and the open pads) and on the entire 2D surface of the plate (**Figure** **4**a). Cell viability was determined using Live/dead fluorescence assay and by Presto blue quantitative assay. Live/dead assay on days 1, 7, and 12 revealed that live cells were homogeneously distributed on the 2D surface of the plate, on the passivation layer of the electrode, and in close proximity to the electrode itself (Figure 4b and Figure S6c,d, Supporting Information). Moreover, the quantitative assay revealed that both the passivated and the bare electrodes did not affect cell viability after 12 days of culture (Figure 4c). In addition, we ensured that the electrical stimulus (50 ms, 3V) through the electrodes did not affect cell viability (Figure S6e, Supporting Information). Taken together, these results indicate the components’ biocompatibility.

Finally, we sought to investigate the ability of the hybrid system, including the electronics and the cell‐containing hydrogel, to collect data from the engineered tissue, and when needed to provide electrical stimulation for pacing the cells. Cardiac cells either derived from iPSCs (Figure S7 and Movies S5–S7, Supporting Information) or neonatal cells were mixed with the ECM hydrogel in its weak state before physical crosslinking, which takes place at 37 °C. As a proof of concept, 20 × 15 × 2 mm^3^ (l/w/h) cardiac patches from neonatal cardiac cells were printed with 8 electrodes (Movie S8, Supporting Information). Six of the inner electrodes were printed with a passivation layer except for their rounded end, leaving exposed pads for point sensing and stimulation. The outer electrodes were completely exposed to provide field‐stimulation to the entire engineered tissue. The electronic patch was soft and could easily withstand handling (Figure 4d and Movie S9, Supporting Information). The patch was cultivated for 7 days and spontaneous contractions could be detected (Movie S10, Supporting Information). Immunostaining of the patches on day 12 revealed that the cells were elongated, expressing high levels of actinin, a protein associated with contraction (Figure 4e).

To evaluate the ability of the printed electronics to monitor the engineered tissue function, the pads at the end of the electrodes were connected to copper wires and an amplifier using a silver paste. As shown, the system could record extracellular potentials from four different locations within the patch in parallel (Figure 4f). The signals exhibited a shape and width characteristic of neonatal ventricular cardiomyocytes (Figure S6f, Supporting Information).^[^ ^29^ ^]^ Then, the system's potential to intervene with the contraction rate of the engineered tissue was demonstrated. Such an intervention may be useful after patch transplantation when the tissue is not contracting properly, not synchronized with the healthy part of the heart, or when other conduction disorders appear in the tissue or the heart. The engineered tissue was placed at room temperature to reduce contractions, and pacing at 1 and 2 Hz was applied through electrical stimulation (7 V, 50 ms long pulses). As shown, the tissue reacted to the external stimulation, contracting synchronously and responding directly to the stimulation regime (Figure 4g). Taken together, these results demonstrate the ability of the printed patch to provide accurate data on its function as well as the ability to control its performance.

## 3 Conclusions

The field of tissue‐electronics interfaces has significantly evolved in recent years. In tissue engineering, electronic systems were integrated with cells to form controllable tissues. However, the electronics were fabricated separately from the tissue, mainly by processes derived from the microelectronics industry such as photolithography. This resulted in limited integration between the electronics, the scaffolding material, and the living part of the patch. In the last several years, the field of tissue and organ printing has immensely advanced. However, since an electronic system could not be printed under physiological conditions, such as 37 °C and aqueous solution, it could not be printed inside an engineered tissue.

In this work, we report the one‐step 3D printing of heart tissue with built‐in electronics. We demonstrate that conductive and dielectric bioinks can be printed together with a cardiac cell‐containing ECM hydrogel. The components of the printed electronics were characterized and optimized. We show that the engineered tissue contracts and the electronics can sense its function and provide electrical stimulation for pacing.

Looking forward, 3D printing has brought to the field of tissue engineering remarkable capabilities. While this technology greatly facilitates the structuring of native tissue‐like architectures, many challenges still remain to be faced (for a comprehensive review of the field see).^[^ ^30^ ^]^ The ability to accurately place different cell types and different electronic components in the 3D space will allow to engineer complex tissues and control their function. Such systems would allow the physician to monitor the function of an engineered tissue after transplantation, and when needed to efficiently intervene from afar. The ability to print whole organs, although still basic in function, would pioneer a new field of bionic organ engineering.

## 4 Experimental Section

##### Materials

PDMS was prepared using sylgard 184 (Dow corning) according to the manufacturer's instructions. Briefly, the base and curing agent were mixed at a 10:1 ratio and degassed in vacuum. Graphite paste was prepared by mixing 45% (wt) graphite synthetic powder (<20 µm, Sigma‐Aldrich) in PDMS. The dielectric ink was prepared by mixing 0.1% (wt) span 80 (Sigma‐Aldrich) in PDMS.

Omenta were decellularized as previously described.^[^ ^22^, ^31^ ^]^ Briefly, pig omenta (Kibutz Lahav–designated for the food industry) were washed with phosphate‐buffered saline (PBS) and then transferred to hypotonic buffer (1 × 10^−2^ m Tris, 5 × 10^−3^ m ethylenediaminete‐traacetic acid (EDTA), and 1 × 10^−6^ m phenylmethanesulfonyl‐fluoride, pH 8.0) for 1 h. Next, the tissues were frozen and thawed three times in the hypotonic buffer. The tissues were gradually washed with 70% (v/v) ethanol and 100% ethanol for 30 min. Lipids were extracted by three 30 min washes of 100% acetone, followed by a 24 h incubation in a 60/40 (v/v) hexane: acetone mixture (the solution was exchanged three times in 24 h). The defatted tissue was washed in 100% ethanol for 30 min and incubated over‐night at 4 °C in 70% ethanol. Then, the tissue was washed four times with PBS (pH 7.4) and incubated in 0.25% Trypsin‐EDTA solution (Biological Industries) over‐night. The tissue was thoroughly washed with PBS and incubated in 1.5 m NaCl (the solution was exchanged three times in 24 h), followed by washing in 50 × 10^−3^ m Tris (pH 8.0), 1% triton‐X100 (Sigma‐Aldrich) solution for 1 h. The decellularized tissue was washed in PBS followed by double distilled water and then frozen (−20 °C) and lyophilized. The dry, decellularized omentum was ground into powder (Wiley Mini‐Mill, Thomas Scientific, Swedesboro, NJ). The milled omentum was then enzymatically digested for 96 h at room temperature with stirring, in a 1 g L^−1^ pepsin solution (Sigma‐Aldrich, 4000 U mg^−1^) in 0.1 m HCl. Subsequently, the pH was adjusted to 7.4 using 5 × 10^−3^ m NaOH in Dulbecco's Modified Eagle's medium (DMEM)/F12 × 10 (Biological Industries). The concentration of decellularized omentum in the titrated solution was 1% (w/v). Then, the 1% (w/v) omentum gel was filtrated using a 70 µm strainer (MTCBio). Subsequently, while constantly stirred, the gel was allowed to reduce under a jet of sterile air until it reached 1/2 of its initial weight. The concentrated gel (2% w/v) was then centrifuged at 300 g for 2 min to remove air bubbles and stored at 4 °C until use.

##### 3D Printing

The printing was performed using a Discovery 3D printer from RegenHu. The bioinks were transferred into 3 mL syringes and centrifuged to remove air bubbles. Then, the inks were simultaneously printed through three extrusion printheads. The graphite ink was dispensed through a 25G conical needle at 5.5 kgf cm^−2^. The hydrogel‐based ink and passivation ink were printed through 27G blunt needles at a low pressure of 1 kgf cm^−2^.

To determine the printing resolution of the graphite ink, the paste was extruded through 27G or 25G tips at different printing rates into 5 lines with varying thicknesses on the glass slide. Then, the printing was visualized using a stereomicroscope (P2‐DBL, Nikon) and quantified.

##### Scanning Electron Microscopy

Hydrogel‐containing samples were fixed with 2.5% glutaraldehyde (24 h at 4 °C), followed by a graded incubation series in ethanol‐water solutions (25–100% (v/v)). Then, the samples were critical point dried. Cross‐section samples were cut using a scalpel. All samples were mounted onto aluminum stubs with conductive paint and were sputter‐coated with an ultrathin (150 Å) layer of gold using a Polaron E5100 coating apparatus (Quorum Technologies, Laughton, UK). The samples were viewed under a JSM‐840A SEM (JEOL, Tokyo, Japan).

##### Electrical Resistance Measurements

Electrical resistance measurements were performed using a DMM‐7510 multimeter (Keithley Instruments, Cleveland, OH) using a two‐probe setup. The conductivity (*σ*) was calculated using the equation: $\sigma \;=\frac{L}{RA}\;$, where *R* is the resistance, *L* is the distance between the probes, and *A* is the cross‐section.

##### Rheological Properties

Rheological measurements were performed using Discovery HR‐3 hybrid Rheometer (TA Instruments, DE) with 8 mm diameter parallel plate geometry and a Peltier plate to maintain the sample temperature. The samples were loaded at a temperature of 24 °C, which was then raised to 37 °C to induce crosslinking, during which the oscillatory moduli of the samples were monitored at a fixed frequency of 0.8 rad s^−1^ and a strain of 1%.

##### Mechanical Testing

To examine the mechanical properties of the graphite paste, dog bone‐shaped structures were prepared according to ASTM D412 98a standard dimensions. The dog bone‐shapes were cut using a mold. To evaluate the mechanical properties of the electrode, core electrodes and passivated electrodes were printed. On each side of the electrode, a pad was printed for holding the electrode in place. All samples were tested for the strain‐to‐break exam using a model LS1 tensile testing instrument (Lloyd Instruments, Ltd.), with a 20 N load cell for the dog‐bone samples and 5 N load cell for the electrodes, at a rate of 5 mm min^−1^. Device control, data acquisition, and processing were performed using the NEXYGEN plus 3.0 software (Lloyd Instruments, Ltd.). The engineering stress was calculated over the length and cross‐sectional area *A* = *W* ^2^, where the initial width (*W*) of the tested area was measured using a caliper for the dog‐bone samples or an inverted microscope (Nikon Eclipse TI) for the electrodes. The engineering stress was calculated as the ratio between the load and the initial cross‐section area of a tensile specimen, while the strain was calculated as the ratio between its extension and its initial length. The Young's modulus for each sample was determined from the slope of a stress‐strain curve. The elongation percentage was calculated as the ratio between the length at the failure point and the initial length.

##### LED Activation

Samples consisting of two passivated electrodes were printed in the hydrogel, crosslinked at 37 °C in an incubator for three days, and then connected to an electrical circuit containing a red LED bulb at one side and a 3V power source (STG 4002, Multichannel systems) at the other side. Thin copper slides were placed in between the printed graphite pads and the electrical clips.

##### Cyclic Stretching and Bending

Samples of core electrodes and passivated electrodes were printed. For cyclic stretching testing, the electrodes were printed with pads on each end on gelatin‐coated slides and detached by washing. The pads were wrapped with copper tape, connected to DMM‐7510 multimeter probes, and placed between the mechanical tester clips. The samples for cyclic bending were printed on a transparent plastic surface with two thin copper strips, which were connected to the multimeter probes through a copper tape. Then, the samples were stretched or compressed 1000 times for stretching or bending tests, respectively, during which the resistance was monitored. Samples after the stretch test were investigated for flaws using SEM.

##### Flexibility Test

Printed samples containing two graphite electrodes in hydrogel and pads on both sides were crosslinked at 37 °C in an incubator for three days. Then, the pads were fixed to a transparent surface and twisted several times till reaching 180°.

##### Patch Formation

Neonatal cardiac cells were isolated according to Tel Aviv University ethical use protocols from intact ventricles of 1‐ to 3‐day‐old neonatal Sprague‐Dawley rats, as previously reported.^[^ ^32^ ^]^ Cells were isolated using 6 cycles (37 °C, 30 min each) of enzymatic digestion with collagenase type II (95 U mL^−1^) and pancreatin (0.6 mg mL^−1^) in DMEM. After each round of digestion, cells were centrifuged (600 g, 5 min) and resuspended in M‐199 culture medium supplemented with 0.6 × 10^−3^ m CuSO_4_ 5H_2_O, 0.5 × 10^−3^ m ZnSO_4_ 7H_2_O, 1.5 × 10^−3^ m vitamin B12, 500 U mL^−1^ penicillin, and 100 mg mL^−1^ streptomycin, and 0.5% (v/v) fetal bovine serum (FBS, Biological Industries). To enrich the cardiomyocyte population, cells were suspended in a culture medium containing 5% FBS and pre‐plated twice for 40 min. Cell number and viability were determined using a hemocytometer and trypan blue exclusion assay. Cell‐laden bioink was prepared by mixing the isolated cardiomyocytes in omentum‐based hydrogel (2 × 10^8^ cells ml^−1^) and transferring into a syringe. After the cellular patches were printed, they were crosslinked in an incubator for 40 min and then M199 medium (Biological Industries) supplemented with 5% FBS was added and replaced every 2–3 days. iPSCs‐derived cardiac patches were printed in the same manner. iPSCs‐derived cardiomyocytes were a kind gift from Prof. Lior Gepstein from the Technion.^[^ ^33^ ^]^

##### Viability Assays

The passivated electrodes were printed on a culture plate in triplicates and rat neonatal cardiac cells were then seeded on top and cultivated at 37 °C. The control group consisted of cardiac cell culture in empty wells.

##### Live/Dead Assay

Cell viability was determined after 12 days of culturing using a Live/dead fluorescent staining with fluorescein diacetate (Sigma‐Aldrich,7 µg mL^−1^) and propidium iodide (Sigma‐Aldrich, 5 µg mL−1) for 10 min at 37 °C. The stained cells were visualized using an upright microscope equipped with a Hamamatsu Orcaflash 4.0 (Hamamatsu) and NIS‐Elements software (Nikon).

##### PrestoBlue Assay

Cell viability was determined on days 1, 4, 7, and 12 of culturing using a PrestoBlue reagent (Invitrogen). The reagent mixture (1:9 in supplemented M199 medium) was added to the examined samples and incubated for 40 min. A sample of the reagent mixture was incubated under the same conditions without cells were used as a blank. Afterwards, the reagent mixture was collected, and absorbance was evaluated in triplicates at 570 nm and at 600 nm as reference. After the mixture was aspirated, the cells were supplied with a fresh medium for further culture. The Viability was calculated as follows: Viability = [(Absorbance_570nm_ – Absorbance_600nm_)_test_ – (Absorbance_570nm_ – Absorbance_600nm_)_blank_]. Normalized viability = (Viability / Viability_control_)

##### Immunostaining

Cardiac patches were fixed after 12 days of culturing using 4% (v/v) formaldehyde for 20 min, washed three times in PBS, and then permeabilized using 0.1% Triton in PBS for 20 min. Then, the patches were washed three times and blocked for 1h at room temperature in 5% bovine serum albumin (BSA) in PBS, after which the samples were washed once. The samples were then incubated with primary mouse anti‐*α*‐sarcomeric actinin antibody (1:200, Abcam) in 0.5% BSA in PBS, for 2h at room temperature. Then, the samples were washed three times and incubated for 2h with Alexa Fluor 647 conjugated goat anti‐mouse antibody (1:250; Jackson, West Grove, PA) and Alexa Fluor 488 conjugated goat anti‐rabbit antibody (1:250; Jackson, West Grove, PA) in 0.5% BSA in PBS. For nuclei detection, the cells were incubated for 10 min with Hoechst 33 258 (1:20 in PBS; Sigma) and washed three times. The samples were embedded in Mowiol solution for preservation, covered with a glass coverslip and a single drop of immersion oil (type N, Nikon) was placed on top. The images were obtained using a scanning laser confocal microscope (Nikon) equipped with oil lenses of x40 and x100 magnifications, and NIS‐Element software.

##### Device Operation

Electrical signals were recorded using X series multifunction data acquisition device (National instruments) and 1700 Differential AC amplifier (A‐M Systems). The samples were placed on a warm surface and connected to the signal recording system. The data was processed to filter the noise by moving average using the Excel software (Microsoft).

Stimulation was performed using an SP‐150 potentiostat (BioLogic, Science Instruments) with a RE‐1B reference electrode (Ag/AgCl). The pacing was performed by applying 7 V for 50 ms‐long pulses at 1–2Hz. Calcium imaging was used to visualize signal propagation.

##### Calcium Imaging

Calcium transients were evaluated as previously described.^[^ ^22^ ^]^ The patches were incubated with 10 × 10^−6^ m fluo‐4 AM (Invitrogen) and 0.1% Pluronic F‐127 for 40 min at 37 °C. Then, the solution was changed to a growth medium and the patches were imaged using an inverted fluorescence microscope. Movies were acquired with a Hamamatsu Orcaflash 4.0 at 100 frames s^−1^ using the NIS‐Element software. Data collected from 3 regions of interest were analyzed using the ImageJ software (NIH). The fluorescence was normalized by dividing the basal cell fluorescence and the first derivative was generated for each data set.

##### Statistical Analysis

All experiments consisted of at least three independent repeats, and the results were expressed as means ± standard deviation (SD). Two‐tailed unpaired *t*‐test with Welch's correction was performed as required using the GraphPad Prism software, and statistical significance was determined at a value of *p*<0.05 for each experiment.

## Conflict of Interest

The authors declare no conflict of interest.

## Supporting information

Supporting Information

Supporting Movie 1

Supporting Movie 2

Supporting Movie 3

Supporting Movie 4

Supporting Movie 5

Supporting Movie 6

Supporting Movie 7

Supporting Movie 8

Supporting Movie 9

Supporting Movie 10

## Data Availability

The data that support the findings of this study are available from the corresponding author upon reasonable request.
