# Supplementary material for: One‐Step 3D Printing of Heart Patches with Built‐In Electronics for Performance Regulation
Source: Adv Sci (Weinh). 2021 Mar 2;8(9):2004205. doi: 10.1002/advs.202004205 (PMC8097332; doi:10.1002/advs.202004205)
Supplement: Supplementary file 1 — Supporting Information [file ADVS-8-2004205-s009.pdf]

© 2021 Wiley-VCH GmbH

ADVANCED SCIENCE
Open Access

# Supporting Information

for *Adv. Sci.*, DOI: 10.1002/advs.202004205

**One-step 3D printing of heart patches with built-in electronics for performance regulation**

Masha Asulin, Idan Michael, Assaf Shapira and Tal Dvir*

**Supplementary information for**

## One-step 3D printing of heart patches with built-in electronics for performance regulation

Masha Asulin[1,2], Idan Michael[2], Assaf Shapira[2] and Tal Dvir[1,2,3,4,*].

[1]Department of Materials Science and Engineering, Faculty of Engineering, Tel Aviv University, Tel Aviv 6997801, Israel.

[2]The School for Molecular Cell Biology and Biotechnology, Faculty of Life Sciences, Tel Aviv University, Tel Aviv 6997801, Israel.

[3]The Center for Nanoscience and Nanotechnology, Tel Aviv University, Tel Aviv 6997801, Israel

[4]Sagol Center for Regenerative Biotechnology, Tel Aviv University, Tel Aviv 6997801, Israel

*Correspondence to Tal Dvir (tdvir@tauex.tau.ac.il)

**This PDF file includes:**

Materials and Methods

Supplementary Figures S1-7

Captions for Movies 1 to 10

**Materials and methods**

**Graphite flakes characterization.** Sample of graphite powder (<20 µm) was placed onto an aluminum stub with conductive paint and was then visualized using SEM. Flakes size and circularity distribution were analyzed using ImageJ (NIH) by measuring 50 particles in three randomly chosen areas. Flakes diameter (d) was calculated as $d= 0.5 \cdot (\pi A)^{½}$, where A is the 2D area of the flakes.

**Confocal laser scanning microscope.** Confocal laser scanning microscopy images of the electrodes were obtained using an Olympus LEXT 4000 confocal microscope (OLYMPUS, Tokyo, Japan).

**Voltage loss measurement.** Voltages of 1V and 3V were supplied using a stimulus generator (STG 4002, Multichannel systems) through the core electrode and passivated electrode and the output voltage was measured using a DMM-7510 multimeter.

**Long-term repetitive electrical stimulation**. Core electrode and passivated electrode were incubated in M199 growth medium (Biological Industries, Beit HaEmek, Israel) and 50-ms-long pulses of 3V at 1 Hz were provided through the electrodes using a stimulus generator for approximately 6h. The resistance was measured before and after the incubation period using the multimeter.

**Long-term function**. The electrodes were incubated in M199 growth medium which was changed every three days for two months. The resistance was measured before and after the incubation period using a multimeter. After the testing, the samples were investigated for flaws using SEM.

**Degradation assay**. The electrodes were incubated in 1 U $ml^{-1}$ collagenase type II (Worthington Biochemical Corporation, Lakewood, NJ) in M199 for seven days. The collagenase solution was changed with a fresh solution every other day. The resistance was

measured before and after the incubation period using a multimeter. Subsequently, the samples were examined for flaws using SEM.

**Cyclic stretching of the electrodes in the hydrogel.** Core electrodes and passivated electrodes were printed surrounded with hydrogel on slides covered with gelatin and crosslinked for 3 days at 37°C. Then, the connecting pads were connected to a multimeter and 30 cycles of cyclic stretching of 20% strain were performed in a humid environment created using a nebulizer. Then, the hydrogel layer was removed from the electrodes to examine the electrodes for flaws using SEM.

**Electrodes toxicity after electrical stimulation.** Cardiac cells, isolated from the ventricles of neonatal rats, were seeded on top of the printed electrodes and cultured in M199 growth medium for 24h. Then, the samples in the experimental group were electrically stimulated at 50 ms-long pulses of 3V at 1Hz for 5 min through the electrodes using a stimulus generator, while no voltage was applied in the control group. Then, cell viability was evaluated using PrestoBlue reagent before the test and 1 h after, as described in the main text. Then, normalized viability was calculated as follows: $\text{viability}_{\text{after test}}/\ \text{viability}_{\text{before test}}$.

**Immunostaining iPSCs-derived cardiomyocytes**

The patches were fixed and stained as described in the main text with primary antibodies against cardiac troponin I (1:100, ab47003, Abcam) and NKX2-5 (1:500, ab91196, Abcam).

**Supplementary figure S1. Characterization of graphite ink**

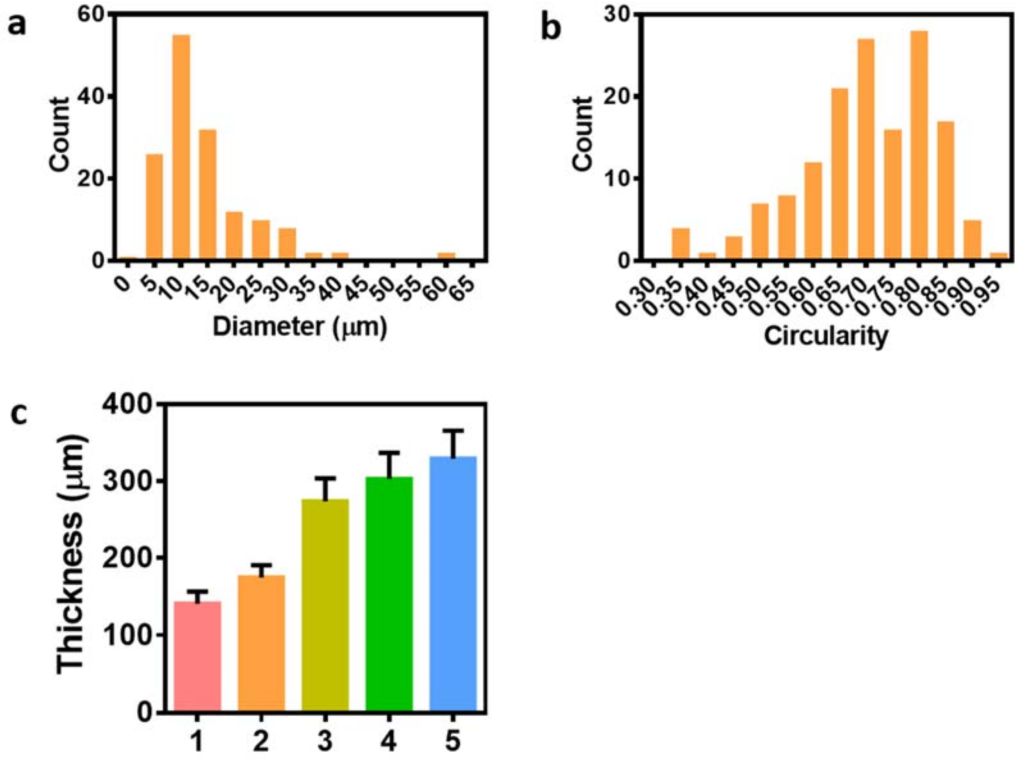


Histograms of (a) the graphite flake diameter and (b) circularity distribution (n=150). (c) Quantification of various electrodes widths presented in Fig. 2d in the main text, from left to right (n=45).

**Supplementary figure S2. Structural and functional characterization of the electrodes**

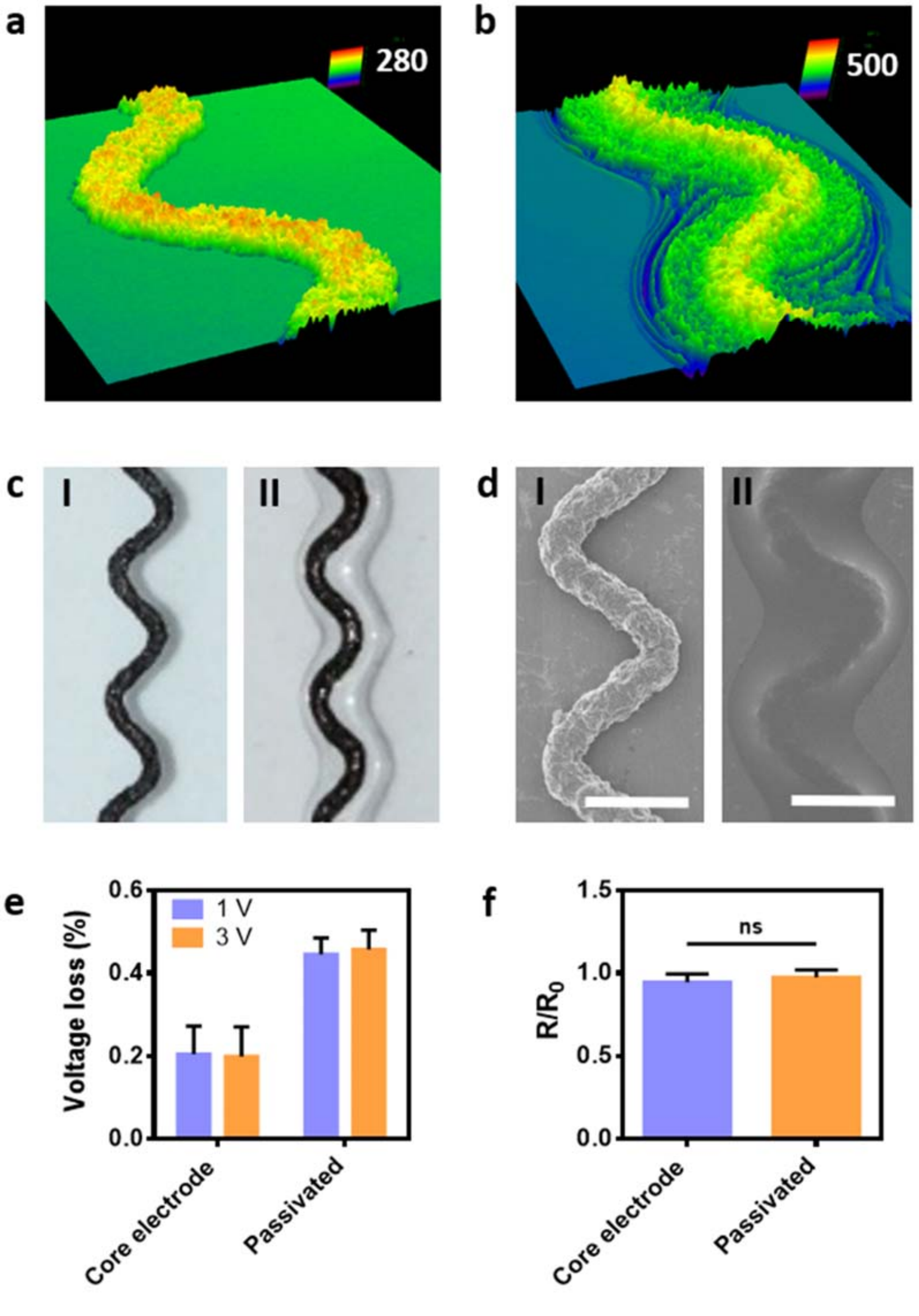


The 3D structures of (a) the core electrode and (b) the passivated electrode were evaluated using the confocal laser scanning microscope. (c) I. Printing pristine serpentine-shape electrode. II. A serpentine-shape electrode with passivation layers. (d) I. SEM image of the

pristine electrode. II. SEM image of the passivated electrode. Scale bar= 1 mm. (e) The calculated voltage loss (%) through the electrodes following the application of 1V and 3V (n=5). (f) The resistance of the electrodes after electrical stimulation of 50-ms-long 3V pulses at 1Hz for 6h, normalized to the resistance before the test (n=3).

**Supplementary figure S3. Effect of degradation on electrode performance and on mechanical properties**

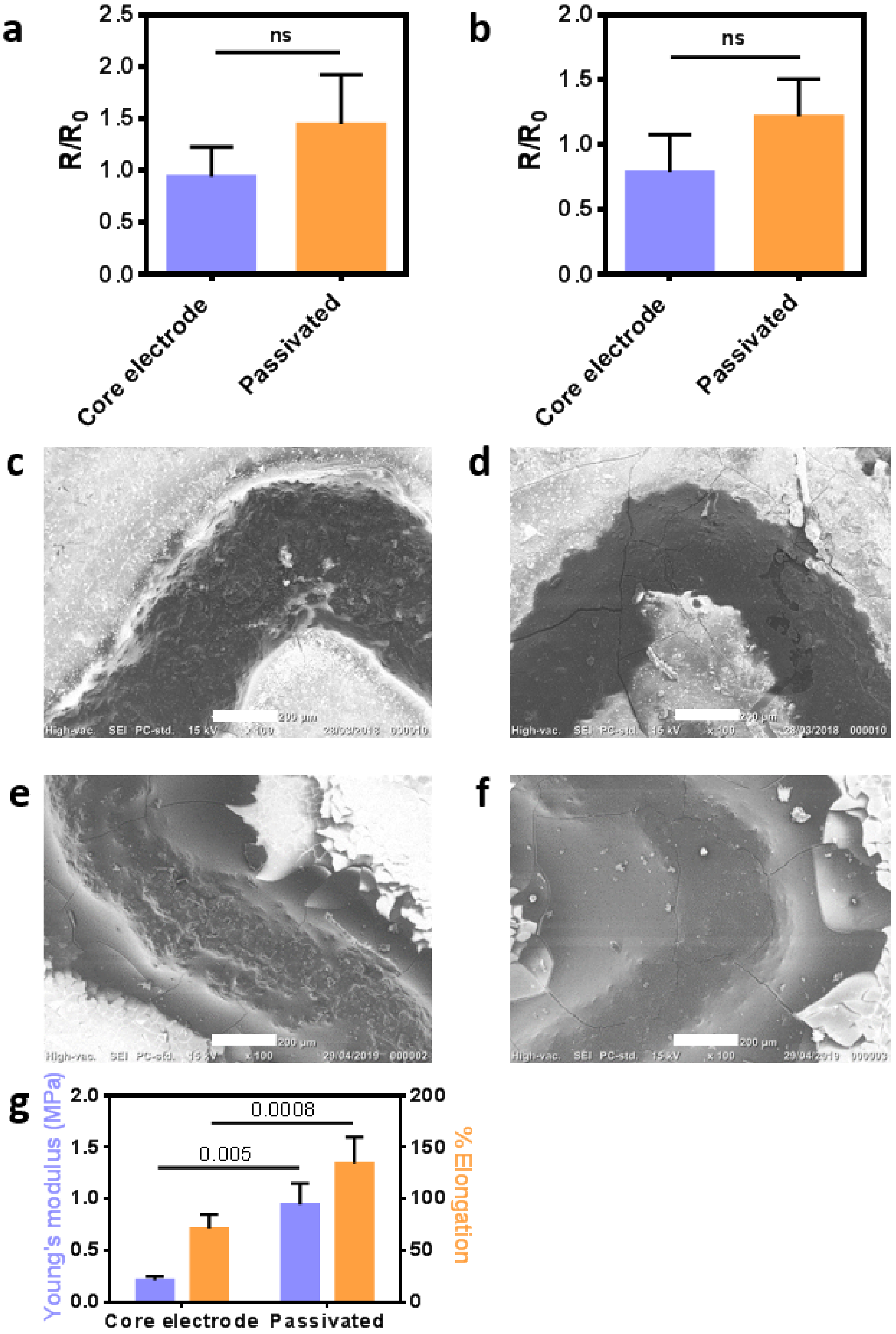

(a) Normalized resistance of the electrodes after two months of incubation in culture medium under physiological conditions. (b) Normalized resistance of the electrodes after a 7-day incubation in collagenase solution. (c-f) SEM images of (c, e) the core electrode and (d, f) passivated electrode. (c-d) Following long-term incubation in culture medium. (e-f) Following incubation with collagenase. Scale bars= 200 µm. (n=3). (g) Young's modulus and elongation of the pristine and passivated serpentine electrodes (n=4).

**Supplementary figure S4. Effect of cyclic stretching and bending on the resistance of the electrodes**

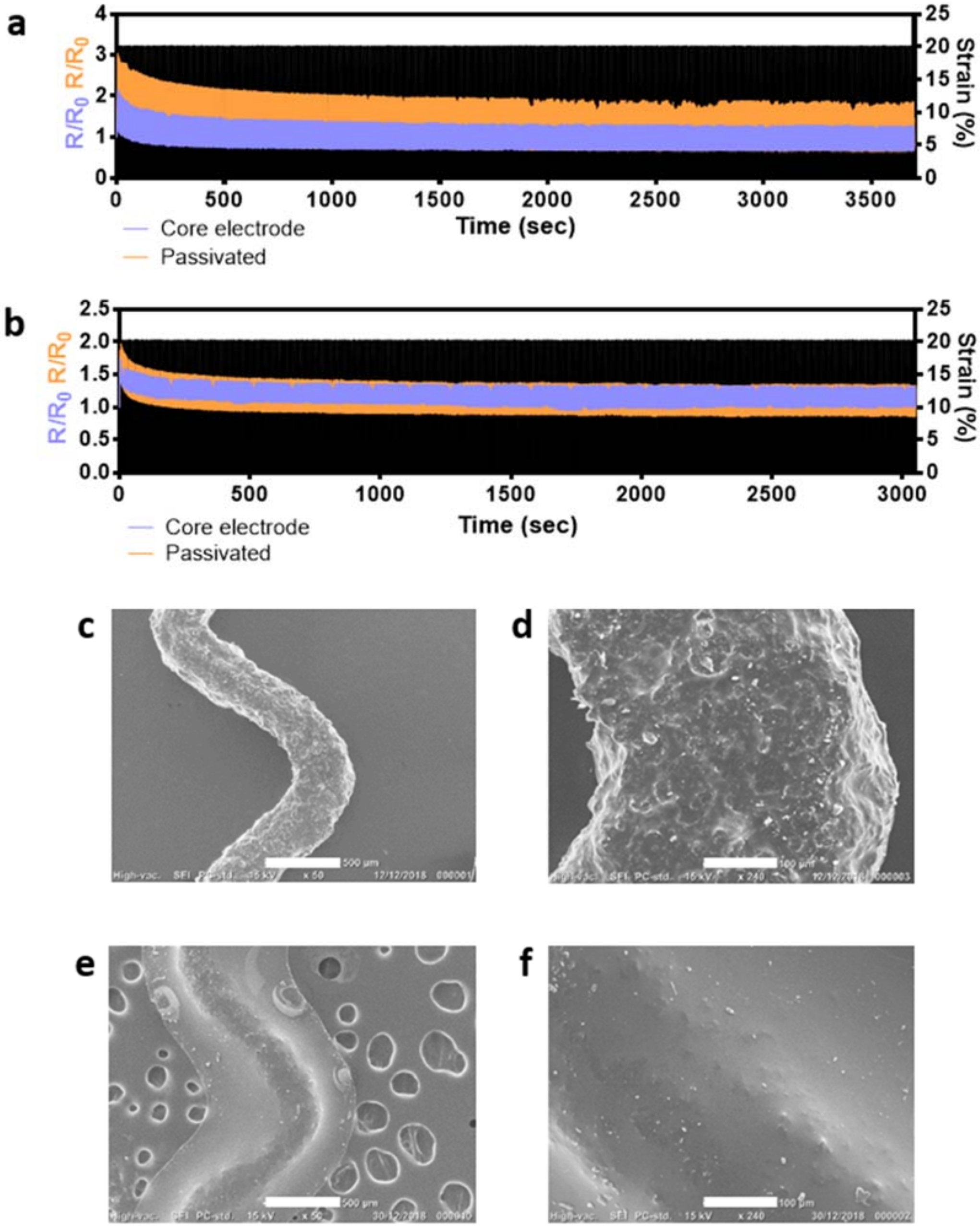


The applied strain and normalized resistance during 1000 cycles of (a) stretching and (b) bending of the electrodes. (c-f) SEM images of (c and d) the core electrode and (e and f) the passivated electrode after cyclic stretching. Scale bars of images c and e: 500 µm and images d and f: 100 µm.

**Supplementary figure S5. Effect of cyclic stretching on the resistance of electrodes in hydrogel**

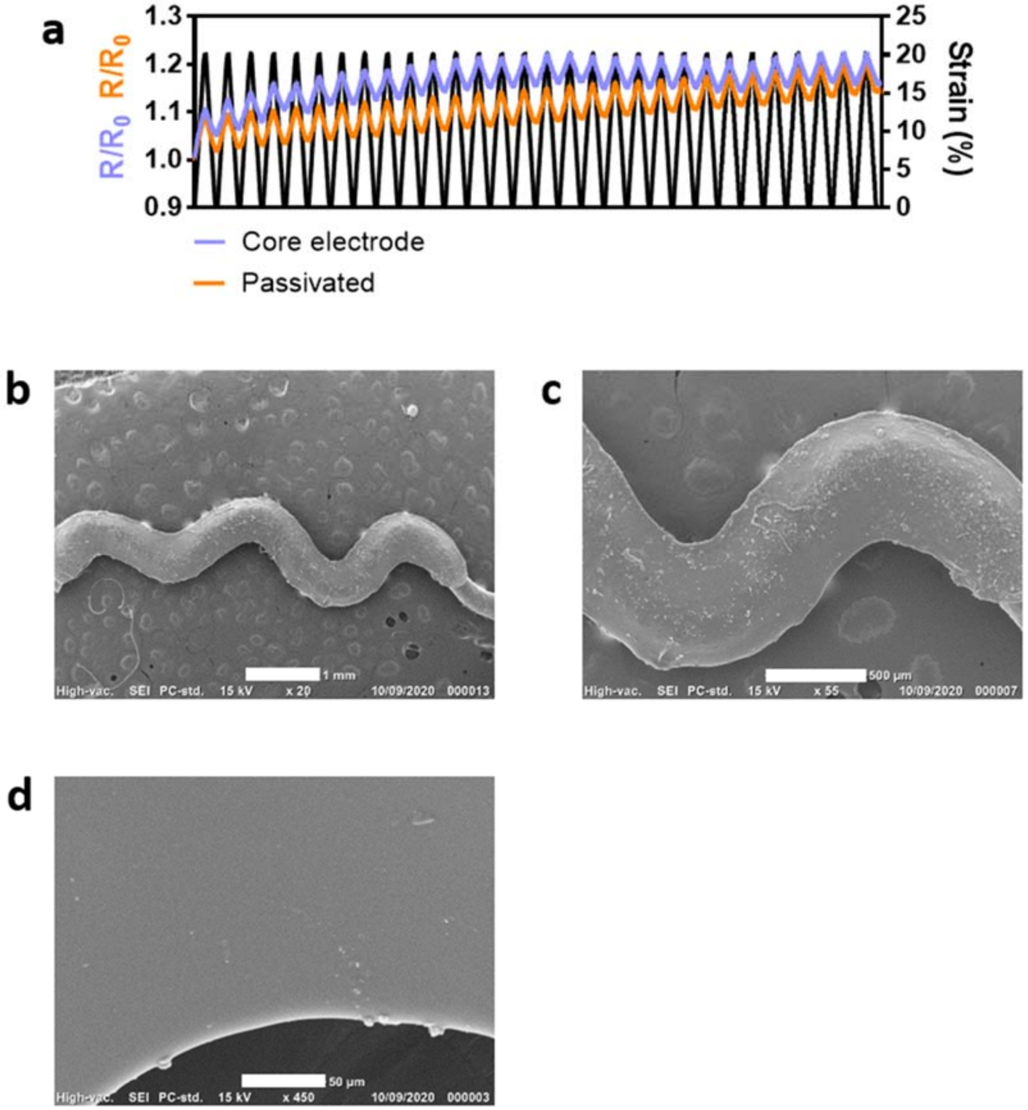


(a) The resistance and strain during 30 stretching cycles of an electrode covered by the hydrogel. (b-d) SEM images of the passivated electrode after cyclic stretching and hydrogel removal. Scale bars of images: b= 1 mm, c= 500 µm, d= 50 µm.

**Supplementary figure S6. Morphology, viability, and patch function**

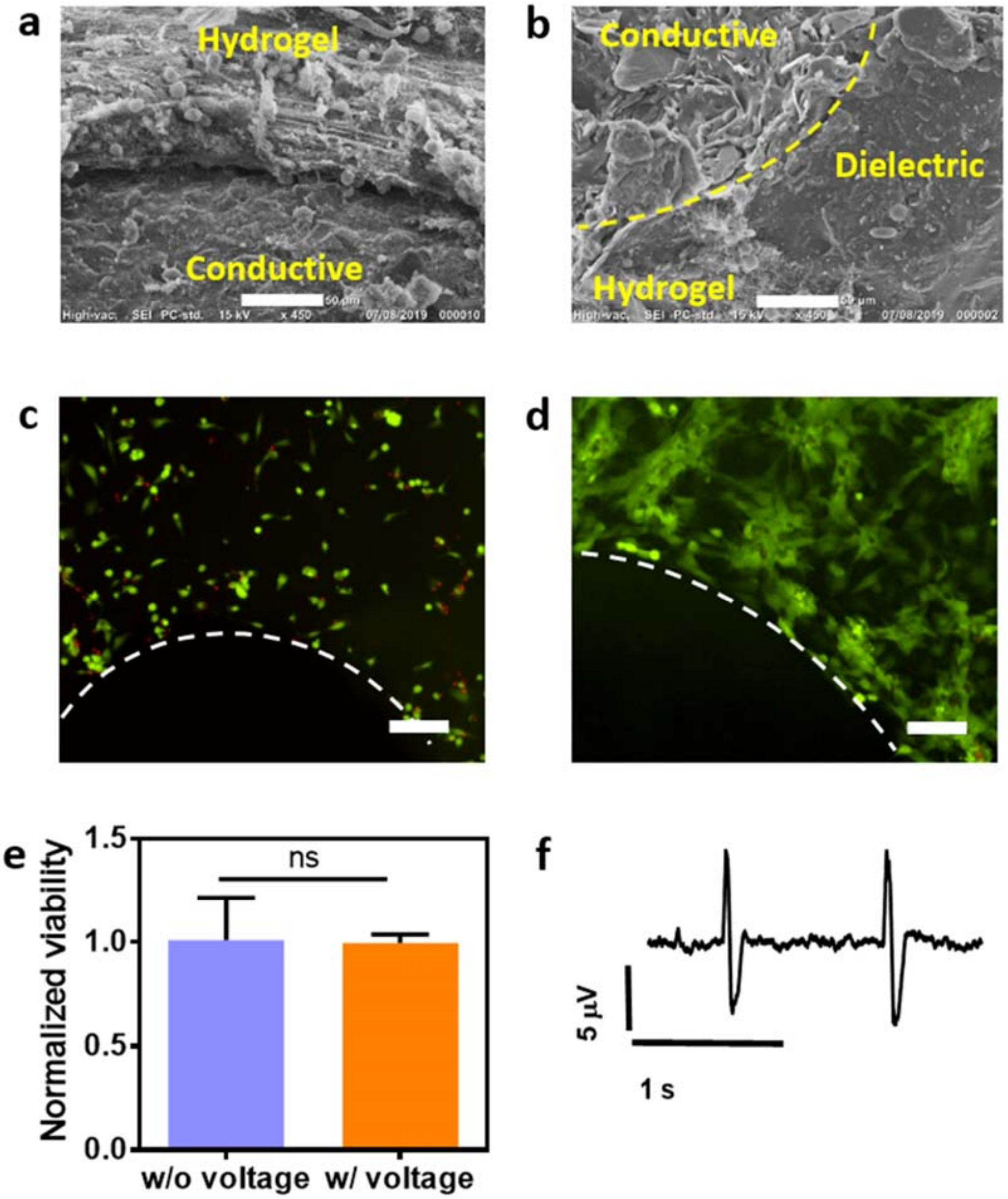


(a-b) SEM images of cell-containing hydrogel – electronics interface on day 7 of culturing. Scale bar= 50 µm. (c-d) Live/dead staining of cardiac cells on and near the electrode (marked by the dashed line) on (c) day 1 and (d) day 7. Scale bar=100 µm. (e) The normalized cell viability following electrical stimulation of 50 ms-long 3 V pulses for 5 min through the printed electrodes (w/ voltage) compared to a control group without external electrical stimulation

(w/o voltage). (n=4). (f). Zoom-in on two representative peaks of the extracellular potentials recording presented in Fig. 4f in the main text.

**Supplementary Figure S7. iPSCs-derived cardiac patch function.**

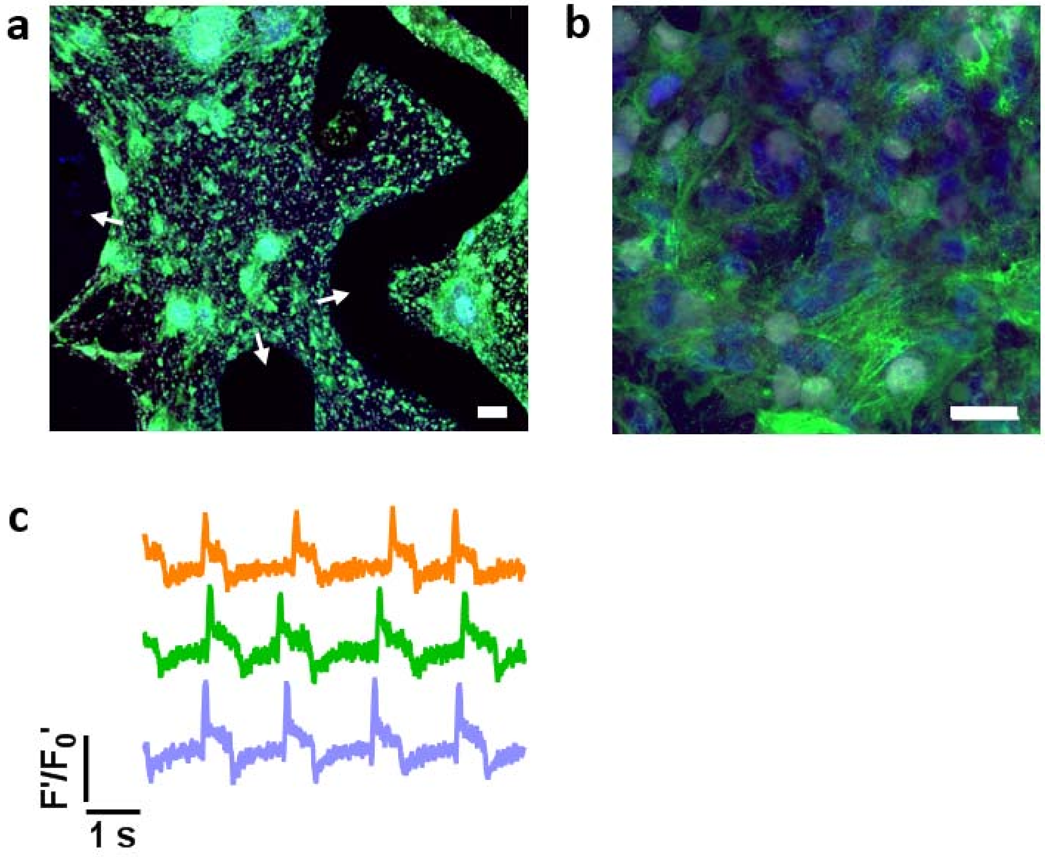


(a-b) Immunostaining of iPSC-derived cardiac patch for troponin I (green), NKX-2.5 (pink) and nuclei (blue) on day 7. Cardiac tissue was formed around the graphite electrodes (arrows). Scale bars: a= 200 µm, b= 20 µm. (c) Calcium imaging of 3 distinct areas within the patch as processed from Supplementary Movie 7.

**Supplementary movies**

**Supplementary Movie 1.**

Strain-stress test of the passivated electrode

**Supplementary Movie 2.**

Cyclic stretching of the passivated electrode

**Supplementary Movie 3.**

Cyclic bending of the passivated electrode

**Supplementary Movie 4.**

Twisting of the electronics-integrated patch

**Supplementary Movie 5.**

Spontaneous contractions of the iPSCs-derived cardiac patch. Scale bar= 100 µm.

**Supplementary Movie 6.**

Calcium imaging of the spontaneous contractions of iPSCs-derived cardiac patch. Scale bar= 200 µm.

**Supplementary Movie 7.**

Calcium imaging of the spontaneous contractions of iPSCs-derived cardiac patch. Scale bar= 100 µm.

**Supplementary Movie 8.**

3D printing of the electronic cardiac patch

**Supplementary Movie 9.**

Electronic patch handling

**Supplementary Movie 10.**

Spontaneous contractions of the cardiac patch. Scale bar= 50 µm.
